# The micro-phenomenology of Floatation-REST

**DOI:** 10.1186/s12906-026-05415-1

**Published:** 2026-05-28

**Authors:** Helena Hruby, Marc Wittmann, Stefan Schmidt, Prisca R. Bauer

**Affiliations:** 1 Institute for Frontier Areas of Psychology and Mental Health, Freiburg, Germany; 2 Department of Psychosomatic Medicine and Psychotherapy, Faculty of Medicine, Medical Center - University of Freiburg, University of Freiburg, Breisgau, Germany

## Abstract

This micro-phenomenological study examines altered states of consciousness (ASC) during Floatation-REST, a sensory isolation method where individuals lie in a tank filled with Epsom salt-saturated water in complete silence and darkness. Salubrious effects of Floatation-REST have been shown in persons suffering from mental health conditions; these positive outcomes might be related to the experience of ASC. Micro-phenomenological interviews were conducted with fourteen participants who completed a 60-minute Floatation-REST session, to capture the generic structure of ASC. We found that most participants experience four distinct phases during a session (diachronic structure): after an initial acclimatization phase (1), participants experience a transitional phase (2) that prepares them for a subsequent ASC phase (3), followed by a phase of temporal and spatial reorientation (4). The ASC phase is characterized by the experience of positive emotions, deep relaxation, a loss of orientation in space and time, and a reduction in bodily sensations and thoughts (synchronic structure). The characteristics of the ASC phase emerge gradually and progressively intensify. This micro-phenomenological analysis of Floatation-REST sheds new light on the potential of this therapeutic technique. We discuss possible future research and therapeutic applications of Floatation-REST.

## Introduction

Floatation-REST involves participants lying in a near-weightless state in a water tank filled with a highly concentrated Epsom salt solution (magnesium sulphate). The water is maintained at skin temperature (35°), and the floating environment (e.g., tank or pool) is quiet and dark. This reduction of external sensory input leads to a decrease in external stimulation, typically resulting in deeply relaxed states.

Previous research has shown that Floatation-REST has beneficial effects on both physical and mental health [1, 2]. These effects have been particularly evident in healthy individuals experiencing stress-related issues [3]. Additionally, clinical studies have demonstrated that it can reduce pain in individuals suffering from stress-related disorders and muscular pain [4, 5]. It has also been shown to alleviate symptoms of generalized anxiety disorder (GAD) [6, 7]. In an early-phase clinical trial with patients suffering from anorexia nervosa Floatation-REST improved anxiety, interoception, and body image disturbances [8].

We have recently shown that Floatation-REST can induce altered states of consciousness (ASC), involving a dissolution of felt body boundaries and a reduction of the sense of time [9]. This research builds upon earlier findings from a Swedish research group, which demonstrated that beyond its relaxing effects, Floatation-REST elicits ASC [10–12]. ASC, such as those induced by meditation or psychedelics, significantly differ from ordinary waking consciousness and are commonly characterized by altered perception of the self [13–17] and time [18–20].

Patients with mental disorders frequently experience an intensified awareness of time. For example, individuals with depression or anxiety frequently report the feeling as though time is dragging or that they are “stuck” in time [21]. Similarly, many mental health conditions are characterized by some form of rigidity or inflexibility [22]. This can manifest on a cognitive level for example as an increase in rumination as this is often seen in depression. It can also manifest on a bodily level. People suffering from depression often perceive their body as heavy, clumsy, almost as an obstacle to life [23]. A meta-analysis on studies using objective movement assessments showed that people with clinical depression or anxiety disorders have less postural control than healthy individuals, and that people with depression tended to show slower gait and reduced step/stride length/width [24]. In contrast, ASC are characterized by a downregulated sense of self and time. The therapeutic benefits observed from interventions that induce ASC, like psychedelics [25] and Floatation-REST [6] may result from their temporary suppression of self- and time-related experiences—specifically, by reducing self-referential processing and diminishing attention to the passage of time. In general terms, ASC induced by such interventions in therapeutic contexts may reduce psychological rigidity and increase flexibility.

Qualitative research approaches to ASC complement quantitative approaches by providing nuanced understanding of such experiences.

One qualitative method to systematically study lived experiences is micro-phenomenology (see below).

It has been applied to various fields, including medical and psychological interventions [26–32], cognitive science and education [32–40], and the arts [41–43]. Only a few studies have applied this method to ASC. To date, it has been used to investigate epilepsy [30] meditative states [15, 44], psychedelic experiences [17, 45], and lucid dreaming [46].

Micro-phenomenology offers the advantage of systematically capturing the subjective experiences of individuals in great detail, allowing for the identification of common experiential features of a singular event across several participants. This is achieved through a retrospective iterative interview process which facilitates the conscious recall of implicit or pre-reflective aspects of experience [47]. Pre-reflective refers to the unconscious dimension of inner experience which precedes conscious thought, reflection, or action. The focus of the investigation is a selected, specific, and temporally limited episode during floating. At the beginning of the interview, participants are guided into an “evocation” of the event under study, involving them to remember, re-experience and re-enact the experience in as much detail as possible.

Following this, a first *diachronic* description of the experience is collected, which is then complemented by *synchronic* descriptions focusing on the quality and structure of the experience (“how” it was lived). *Diachronic* refers to the fine-grained temporal structure of subjective experience across time, while *synchronic* refers to the description of the internal structure of an experience at a given moment. The latter focuses on what is present simultaneously within a single experiential episode. The interviewer alternates between diachronic- and synchronic-orientated questions, frequently paraphrasing the participant’s responses to ensure accurate understanding and deepen the evocation to retrieve more fine-grained material. The interview process is non-linear and progressively refines the description through iterative exploration. The interview is completed when the interviewer gives a comprehensive summary of the experience and the interviewee is satisfied with this description and has no new information to add.

To date there is no systematic study of the subjective experience of ASC during Floatation-REST. How do people experience the ASC during Floatation-REST? How do they enter this state, and how do they come out of it? To answer these questions, we conducted micro-phenomenological interviews with 14 participants after a one-hour floating session to gain insight into the specific experiential dynamics of ASC during Floatation-REST.

## Methods

### Experimental design and sample

This study employed micro-phenomenological interviews to explore and analyze the subjective experience of ASC during Floatation-REST. The initial sample size of eight participants was expanded progressively until data saturation was achieved at *N* = 12. To confirm data saturation, an additional two participants were included. The final sample included 14 healthy adult participants (7 male and 7 female participants). Two of the participants were interviewed a second time to enable internal validation of the findings.

The convenience sample of 14 participants was recruited through word of mouth and through a leaflet, as participants recommended the study to other individuals. All participants completed three Floatation-REST sessions on separate days. The first two sessions (30 min and 60 min duration) were acclimatization sessions to reduce novelty effects and to ensure people were familiar with the Floatation-REST procedure. The third session was the experimental session (60 min duration), which was immediately followed by a micro-phenomenological interview with a length of approx. 60 min. Prior to the first session, participants received detailed written information outlining the study procedure and data protection regulations. At the start of the first session, this information was discussed with the participants and they had the opportunity to ask questions, after which they provided written informed consent. Demographic data were collected, including participants’ age, number of previous floating experiences, and their practice of contemplative techniques. Prior to each session, participants received oral and written instructions on safe floating procedures. A checklist identifying potential health risks was completed and signed before each session.

The interview at the end of the third session was conducted in a quiet, separate room. Following oral consent, interviews were recorded using a digital audio device (Tschisen; MP3/WAV format). Transcripts were pseudonymized and subsequently analyzed.

## Floatation-REST

The Floatation-REST sessions were conducted in the Prana Health Practice in Freiburg, Germany, in the “Cabin for Two” floatation cabin manufactured by Floataway (Norfolk, United Kingdom). The cabin comprises a float tank measuring 180 cm in width and 237 cm in length, located in a private room, which is also equipped with a shower. The tank contains approximately 1,100 L of water enriched with 550 kg of Epsom salt (magnesium sulfate), resulting in a water depth of 27 cm. Both the water and air temperatures were regulated to match skin temperature (~ 35 °C) to minimize tactile sensory input. The total alkalinity of the solution was maintained at 150 ppm, with pH levels fluctuating slightly between acidic and neutral values (6.8–7.2). The consistent saltwater density of 1.25 enabled effortless flotation through the felt buoyancy, with approximately one-third of the body remaining above the water surface while the rest was submerged.

Participants changed clothes and showered under standard lighting conditions. Once inside the tank, the room lights were turned off. Participants retained control over the in-tank lighting through a switch, allowing them to switch the light on or off as desired. A safety button was also available, which triggered an acoustic alarm if needed.

## Micro-phenomenological interview

The interviews were conducted in German by the first author using the micro-phenomenological method. The entire interview process and analysis were based on the guidelines set out by Petigmengin [47]. The interviewer, who holds a Master’s degree in psychology, received formal training in the interview technique from Claire Petitmengin in December 2022, and subsequently completed training in the analysis procedure with Camila Valenzuela-Moguillansky between February and March 2024. Prior to the interview, all participants were thoroughly informed about the structure and purpose of the interview, including the aim of exploring ASC. The interviewer explained that participation was entirely voluntary, that the interview could be interrupted or terminated at any point, and that participants could decline any questions they felt were too personal.

**Fig. 1:**
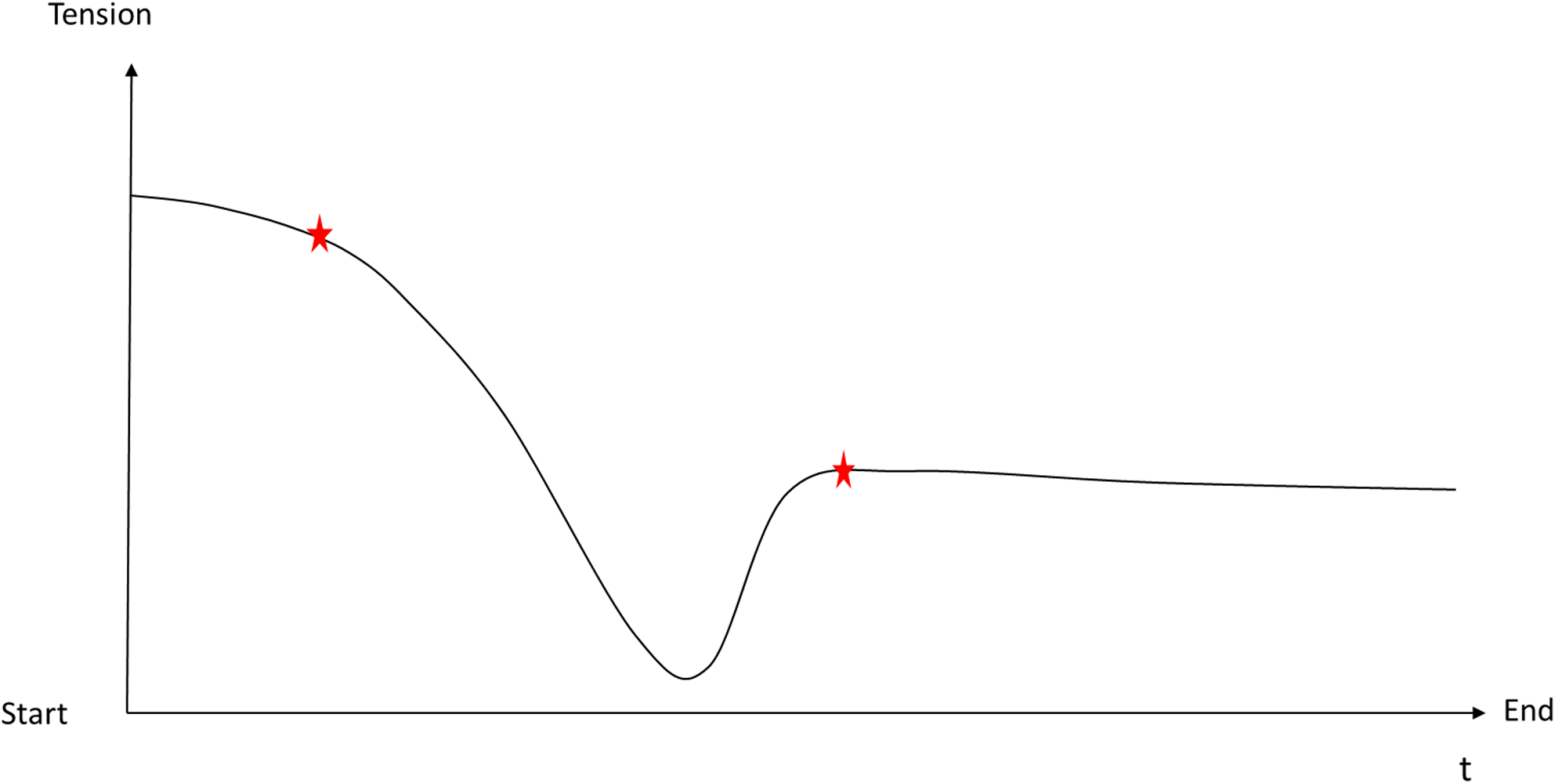
Curve of the floating episode. Floating curve with the tension scale on the y-axis. The asterisk represents the beginning and end of the selected episode. The x-axis represents the time course of the floating session from start to finish (‘t’ = time)

To initiate the interview process, participants were asked to identify a significant moment of ASC during their 60-minute Floatation-REST session by drawing a continuous curve on a blank sheet of paper representing their subjective experience throughout the entire 60-minute floating session. A horizontal arrow served as a fixed timeline, while the vertical axis could be freely labeled by the participant. As an example, the vertical scale might span from “tension” to “relaxation,” illustrating fluctuating experiential intensities, as seen in Fig. 1. Participants then explained their individual curve. and were asked to if there was a particularly salient or meaningful experience (including ASC). The interview then focused on identifying transitions or moments of experiential change, particularly around the onset and resolution of the ASC. To define the boundaries of the selected episode, participants marked the beginning and end with crosses or asterisks on their curve (see Fig. 1). To aid recall, the interviewer guided participants into a focused state of recollection by prompting them to mentally return to the onset of the selected episode. This evocation process was facilitated through targeted questions about sensory details such as body position in the float tank, ambient sounds, smells, or visual impressions. Once the participant had re-accessed this experience and was able to articulate its details, an initial diachronic (temporal) description of the episode was obtained. This was followed by a more detailed exploration of specific experiential elements through both diachronic and synchronic (qualitative) questioning. Paraphrasing and repetition were used to ensure mutual understanding and precision in capturing the participant’s experience. The interview concluded with a chronological summary of the most significant experiential moments within the selected episode, which the participant was asked to correct or add information to. The interview was concluded when no new information was added by the participant.

## Transcription and analysis

The transcription software f4transkript (audiotranskription, dr. dresing & pehl GmbH) was used for the manual transcription of interviews 1 through 11. The remaining interviews were transcribed using the program Subtitle Edit (https://www.nikse.dk). The resulting text files were subsequently revised using f4transkript. During the transcription process, grammatical errors were corrected, and specific linguistic features were encoded: interruptions by another person (= =), sentence breaks by the speaker (-), overlapping speech between both interlocutors (//), and pauses (up to three seconds: (…), longer than three seconds: (number). To facilitate the subsequent analysis, line numbers were added to the transcribed material.

The micro-phenomenological analysis aims at an in-depth investigation of individual experiences within interviews (“specific analysis”) as well as the derivation of a higher-order structure across multiple interviews (“generic analysis”). This analysis follows an iterative process comprising several steps, guided by the framework provided by Valenzuela-Moguillansky and Vásquez-Rosati [48]. All steps in the analysis were reflected regularly in a small team of senior researchers with a background in neuroscience and psychology (all co-authors), and supervised by the last author who has a background in medicine, psychotherapy and extensive experience in applying the method since training in 2016 with Claire Petitmengin. This triangulation of researchers combined multiple perspectives to enhance the validity, reliability and depth of the research findings. The analytical procedure is described in detail in the subsequent section.

1. Preparation

Each interview was checked using a micro-phenomenological quality checklist. Relevant textual elements describing the direct experience of the event were extracted and retained for further analysis, while other information such as general descriptions or reflective commentaries which do not pertain directly to the experience itself (“satellite information”) were excluded from analysis. Since the interviews were not video recorded, paraverbal elements such as gestures were not considered. However, speech pauses were documented through the audio recordings.

2. Specific analysis

In the specific analysis, a distinction is made between *diachronic* and *synchronic* analysis of individual interviews. The first analytical step is the *specific diachronic analysis* and involves the extraction of minimal units of meaning (“descriptemes”) that describe the event. These units are organized chronologically and grouped into overarching phases or subphases. Each diachronic unit is assigned a concise label, and the criteria for temporal grouping are documented. The diachronic structure of each interview is then visualized using a mind map to illustrate the temporal fragmentation of the experience.

The *specific synchronic analysis* focuses on the content characteristics of the identified phases. As this study focuses on ASC, the relevant ASC phases were chosen for in-depth analysis. A bottom-up approach was used for categorization, whereby increasingly abstract categories are formed from minimal units of meaning within a given phase. Two abstraction techniques are applied: *fragmentation*, in which various subcategories are grouped into a broader category, and *specification*, which describes specific features of a higher-level category. To enhance clarity, the synchronic structures of each interview are visualized in the form of semantic networks, which illustrate the relationships between categories and units of meaning.

3. Generic analysis

In the *generic diachronic analysis*, phases across multiple interviews from different participants are synthesized. Phases with similar features are grouped and labeled accordingly. The resulting overarching phase structure is presented and described as a central finding. The *generic synchronic analysis* aims to consolidate all content-relevant information from the semantic networks of individual interviews into a meta-network. This meta-network highlights the central categories and depicts their interrelationships using arrows. The results section is based exclusively on the generic analysis, thereby illustrating a coherent structure of subjective experiences across participants. Findings from the specific analysis are not presented in the results section.

## Results

Fourteen participants completed the study, seven men and seven women (mean age: 29.7 years). All participants were fluent in German. Ten participants held a university degree, three had completed the equivalent of a high school diploma, and one had a secondary school certificate. One participant had one prior experience with Floatation-REST, seven had experienced two floatation sessions, three had experienced three sessions, and three had attended five previous sessions. All of them had a background in regular contemplative practices such as meditation, yoga and chi gong: three on a daily, five on a weekly, and six on a monthly basis.

### Generic diachronic analysis

**Fig. 2:**
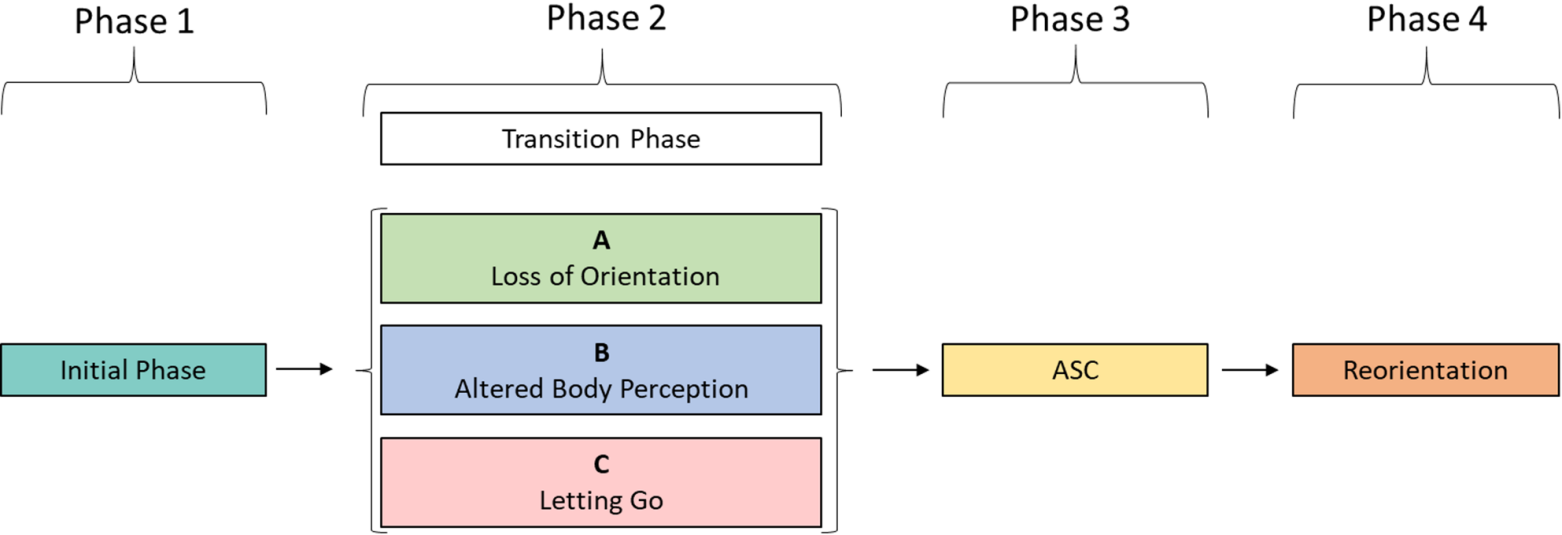
Generic diachronic analysis

**Fig. 3:**
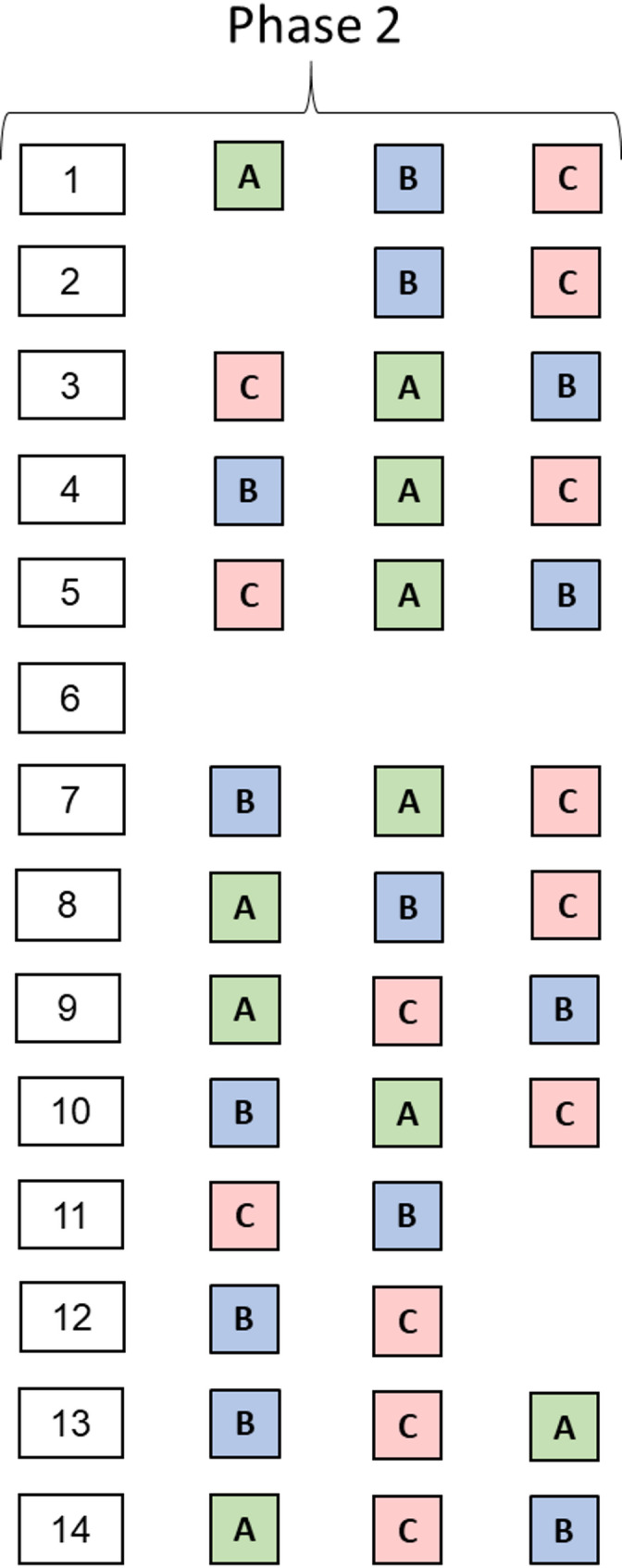
General diachronic structure of *Phase 2: Transition phase*. Note: Illustration of the structure and sequence of the three sub-phases of transition phase *2 A: Loss of orientation*, *2B: Altered body perception*, and *2 C: Letting go*. The visualization depicts the individual order of these subphases for each of the 14 conducted interviews (from left to right). In Interview 6, no *Phase 2: Transition Phase* was identified. Besides the sequential progression of the subphases, overlaps between them are also possible

The analysis of the overarching diachronic structure of a floating episode revealed four phases (see Fig. 2): *Phase 1: Initial Phase*, followed by *Phase 2: Transition Phase*, which is further divided into three subphases: *2 A Loss of Orientation*, *2B Altered Body Perception*, and *2 C Letting Go*. These subphases can occur in varying sequences (see Fig. 3). The third phase, *Phase 3: ASC*, encompasses the experience of ASC, followed by the fourth and final phase of “Reorientation” (*Phase 4: Reorientation*) which may occur spontaneously, or it may be triggered by the light and gong that signal the end of the flotation session. Each of the four phases is described in detail below and illustrated with representative quotes from the interviews. The quotes were translated from German into English for this purpose by the authors. The quotations are referenced using the participant number (e.g., participant 1) and the exact line number from the interview transcript (e.g., line 1).

*Phase 1: Initial Phase* refers to the participants’ initial condition in the floating cabin. Participants describe, among other aspects, the physical position and posture within the tank, as well as their emotional and cognitive state and sensory perception during this early stage in the floating cabin. The participants report a clear perception of their bodies, including distinct awareness of bodily boundaries. From an emotional perspective, this phase is generally experienced as pleasant and relaxing. However, unpleasant sensations such as feelings of effort and restlessness also occur. Additionally, some participants report experiencing a sense of pressure or expectation to relax.

“*My arms were also very relaxed and lay on the surface of the water with the palms facing upwards*” (participant 1, line 157).

*“Um (.) So I was very aware of my body and where my body ends or where my skin begins*.” (participant 9, line 97).

“*And then at the beginning I was somehow*,* I was also relatively agitated*,* I realized that I wasn’t that calm and somehow the most diverse thoughts came to my mind*,* also about topics that were somehow stressing me at the time*,* and that came up first*” (participant 14, line 60).

”*Somehow I had the feeling that there were a lot of thoughts and at some point the moment came when I was thinking a lot and then there was an expectation*,* okay*,* now something has to happen and now do it properly and*,* um*,* (.) yes*,* where there was such an effort. Um*,* exactly*,* and then I somehow also felt (.) a tension in my thighs*“ (participant 5, line 271).

The *Phase 2: Transition Phase*, comprising three subphases A, B, and C, can be interpreted as the transition leading into ASC (*Phase 3: ASC*). The subphases *2 A: Loss of Orientation*, *2B: Altered Body Perception*, and *2 C: Letting Go* occur in varying sequences (see Fig. 3).

In subphase *2 A: Loss of Orientation*, participants report loss of spatial orientation, they can no longer clearly determine their position within the floating tank. This phenomenon is often accompanied by a subjective sensation of upward movement or floating. Additionally, this phase frequently involves a loss of the sense of time, complicating temporal orientation.

“*Yes. Timeless*,* […] but there was also a moment when I thought*,* so*,* yes*,* it’s not quite clear now whether I’m lying somehow-. So*,* gravity was suspended*,* I had-. That - well*,* yes. That in the sense of spaceless. So*,* I thought*,* yes*,* I can also stand*,* float*,* float standing. Floating on my stomach*,* floating on my back*.” (participant 5, line 2016).

Subphase *2B: Altered Body Perception*, within *Phase 2: Transition Phase*, includes all subjective sensations related to one’s own body, encompassing interoceptive experiences. Characteristic phenomena of this phase are the dissolution of perceived body boundaries as well as the subjective experience of enhanced physical mobility. These changes are perceived positively by most of them.

“*And (…) at that moment*,* somehow*,* um*,* my body became a bit blurred with the water. So again*,* this extreme blurring of boundaries*,* yes.*” (participant 2, line 214).

The subphase *2 C: Letting Go* describes a psychological process of releasing and surrendering that initiates a state of relaxation. Participants report consciously relinquishing control and a sensation of falling. This experience is often accompanied by the sensation of being supported and held by the water, which fosters a deep trust in the environment and promotes surrender to the state.

“*(17) Hm (…)*,* yes*,* it’s um*,* (6) yes*,* going along with it. So me*,* kind of surrendering to the water*,* so that the water can carry me*,* um (6) yes and so a bit of this*,* um*,* yes*,* me*,* letting myself drop in there*,* but it’s not letting myself drop*,* but it’s (…)*,* yes*,* entrusting myself to it (…) like that.*” (participant 8, line 365).

*Phase 3: ASC* is described as a stable trance-like, deeply relaxed state. It is clearly distinct from ordinary waking consciousness as well as from the preceding phases. Perceptions of space and time are largely suspended. Participants report a reduction in the number of thoughts, sometimes leading to a content-free state of awareness. They report either neutral experiences or positively feelings such as a sense of safety, inner satisfaction, connectedness, or happiness. The perception of one’s own body is markedly diminished; bodily boundaries recede into the background or dissolve entirely, and physical sensations are scarce. Additionally, participants describe luminous phenomena (luminescence experiences), such as perceiving an energetic aura surrounding the body.

“*[…] And I saw*,* (…) I don’t know*,* it’s difficult with my eyes closed*,* (…) but I felt-*,* I felt*,* (…) ah I’m just fully back again*,* (…) like such bright points of light-*,* (…) and also*,* also*,* I*,* well*,* at least a part of me consisted of this light.*” (participant 3, line 160).

*Phase 4: Reorientation* involves the restoration of temporal and spatial orientation. This phase either occurs very spontaneously and suddenly or is initiated by the light and gong. During this phase, both bodily boundaries and physical sensations become clearly perceptible again. Participants report an increased sense of presence and a conscious contact with themselves. Often, a state of physical vitality or the feeling of being energetically “recharged” is described. On a cognitive level, there is a return to everyday thought processes, accompanied by an initial conscious reflection on the preceding experiences.

“*It just felt like falling from this light or falling back into the body*,* and (4)*,* yes*,* I was just more*,* (…) more aware of myself and*,* oh*,* I’m (…) here in the floating cabin and (…) my thoughts became completely different again and I registered what was happening and tried to unravel it and (…) reexperienced what happened*” (participant 3, line 553).

## Generic synchronic analysis

**Fig. 4:**
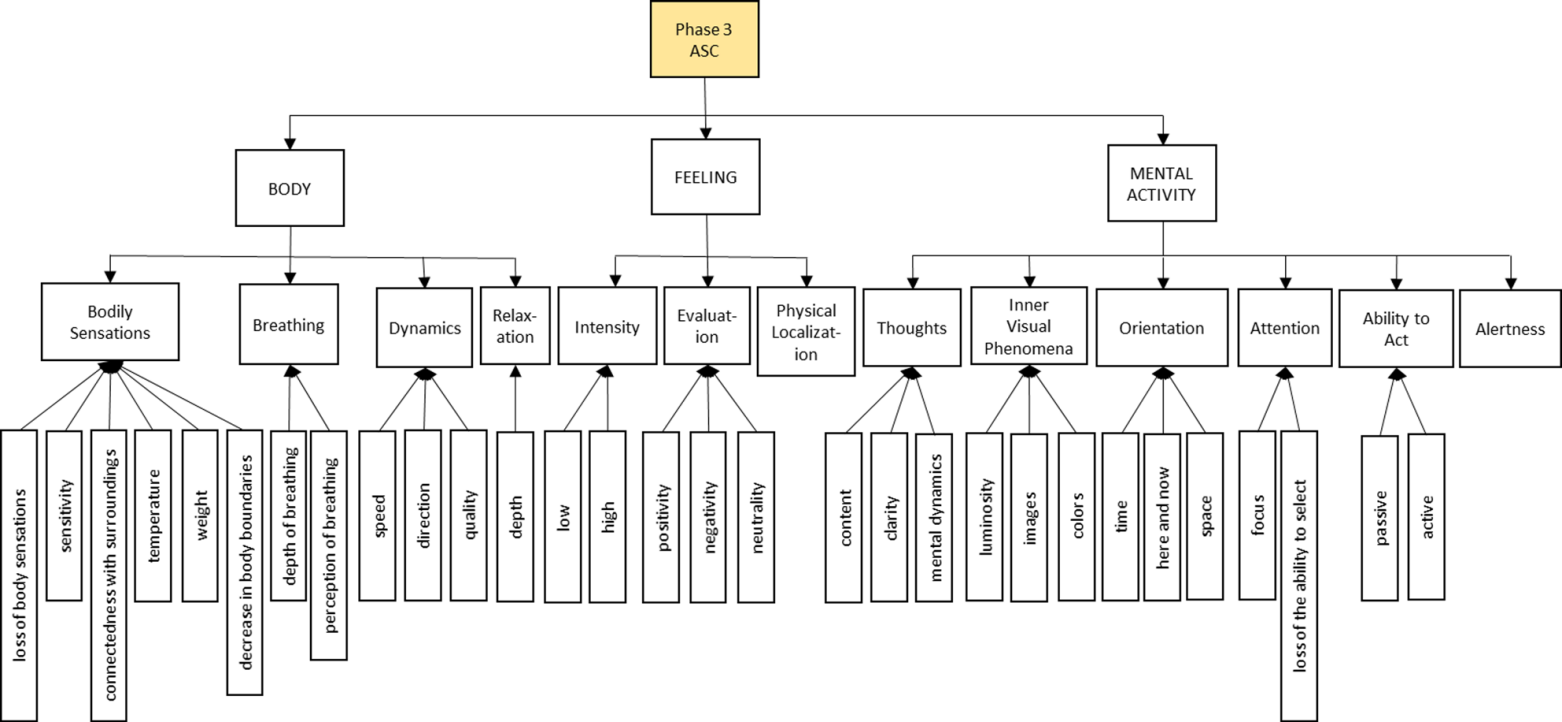
Generic synchronous analysis: Semantic network for the overview of *Phase 3: ASC*

**Fig. 5:**
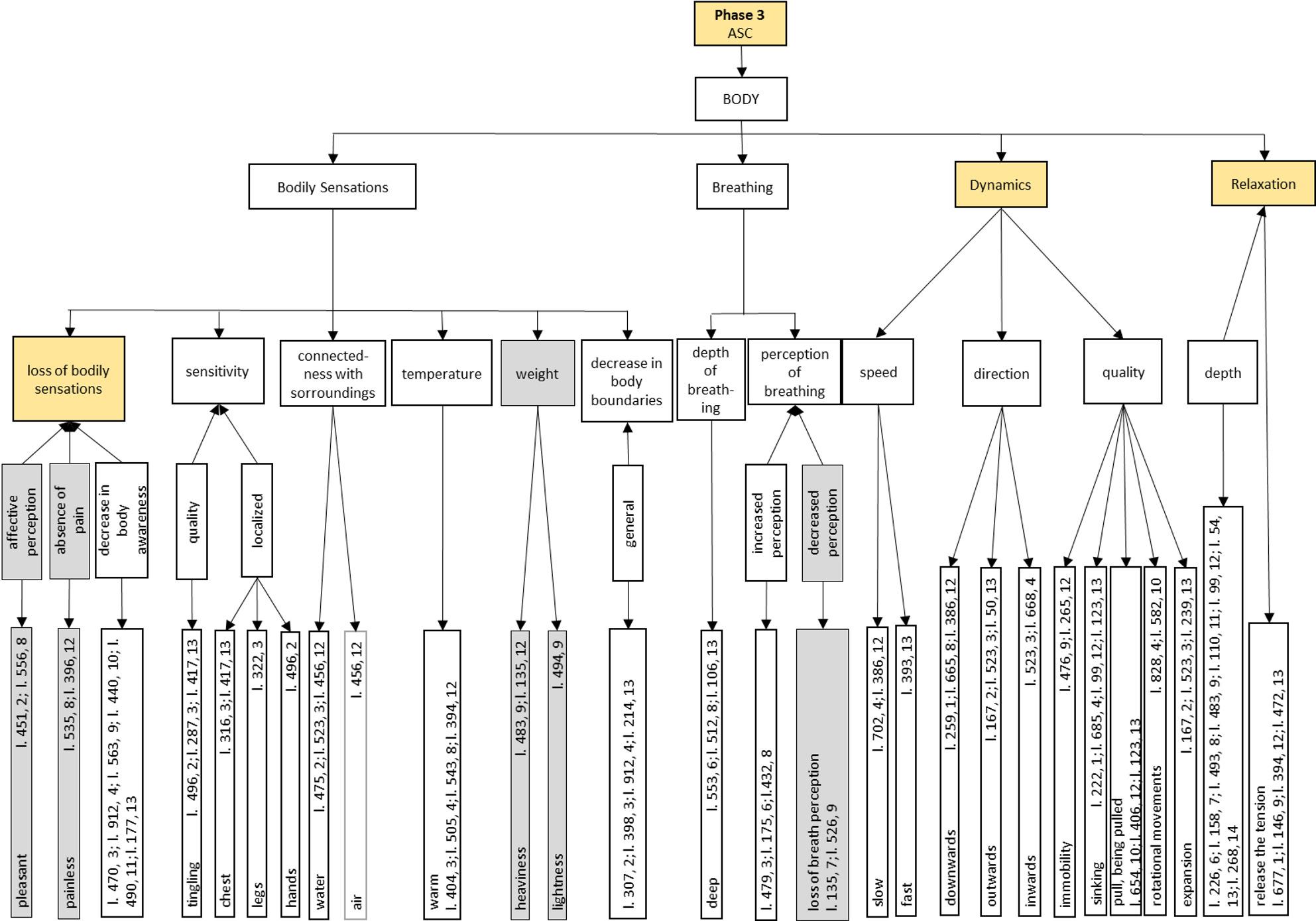
Generic synchronous analysis: Semantic network of Phase 3: ASC on physical aspects. Based on the quotations of the units of meaning, which are indicated by the line numbers of the interview transcriptions and the corresponding participant ((line) l. 34, (participant) 1), main categories were formed through several abstraction steps. The resulting levels of the categories are connected by arrows. Arrows running from top to bottom indicate the abstraction step of fragmentation. Whereas arrows pointing in the opposite direction represent specification. All categories that were cited seven times or more (at least in 50% of participants) are marked by the corresponding color of the phase and thus form a characteristic feature of the respective phase. If the categories were cited less than three times, they are highlighted in gray. In this figure, the following categories are highlighted in yellow: Loss of bodily sensations, Dynamics, and Relaxation. The categories weight and decreased perception are shaded in grey

**Fig. 6:**
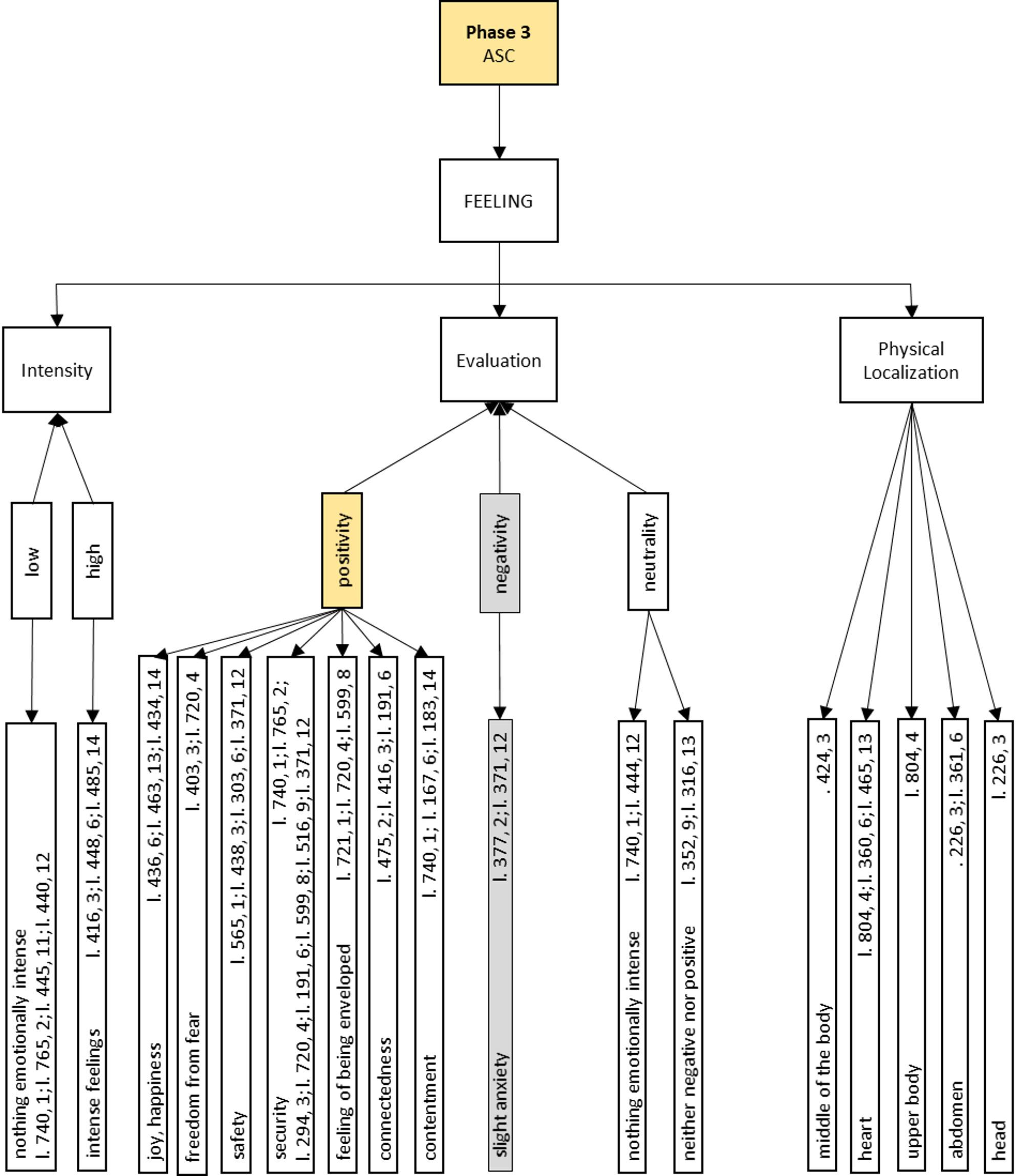
Generic synchronous analysis: Semantic network of Phase 3: ASC on emotional aspects. In this Figure, the category positivity is highlighted in yellow. The category negativity is shaded in grey. For further details concerning the construction of this Figure see Figure 5

**Fig. 7:**
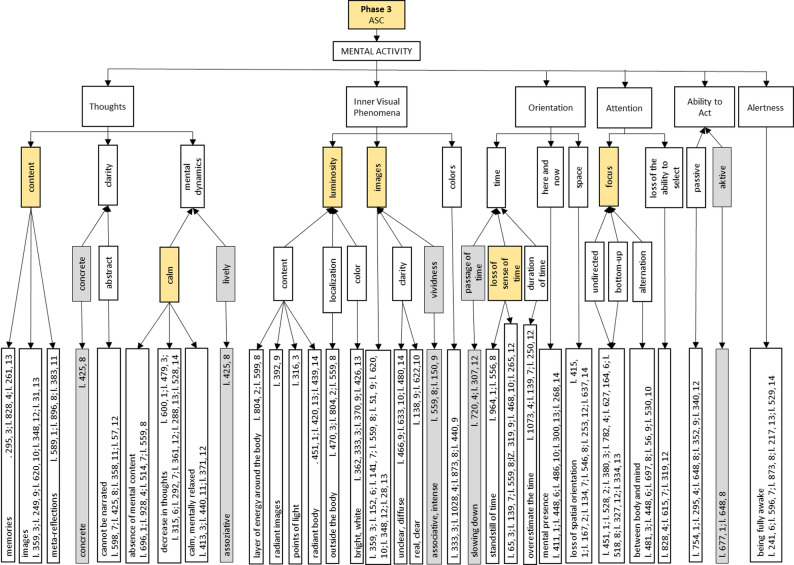
Generic synchronous analysis: Semantic network of *Phase 3: ASC* on cognitive aspects. In this Figure, the following six categories are highlighted in yellow: *Content*, *calm*, *luminosity*, *images*, *loss of sense of time* and *focus*. The categories *concrete*, *lively*, *vividness*, *passage of time* and *active* are shaded in grey. For further details concerning the construction of this Figure see Fig. 5

In the following we present the semantic networks of the generic synchronic analysis of *Phase 3: ASC*, as they offer direct insight into our research question. The semantic networks are structured hierarchically and shown in Figs. 4, 5, 6 and 7.

## Generic synchronic structure of phase 3: altered state of consciousness

The analysis of this phase sheds light on the research question of this study, i.e. what exactly happens during an ASC induced by Floatation-REST. This phase can be divided into the main categories of BODY, FEELlNG and MENTAL ACTIVITY (see Fig. 4). Each of the three main categories is presented in more detail in its own semantic network in Figs. 5, 6 and 7.

The main category BODY has four subcategories *Bodily Sensations*, *Breathing*, *Dynamics* and *Relaxation*. The *Bodily Sensations* category refers to the subjective perception of one’s own body and can be further subdivided into the following aspects: *Loss of body sensations*, *sensitivity*, *connection with the surrounding environment*, *temperature*, *weight* and *decrease in body boundaries*. The category of *Breathing* comprises the two subcategories of *depth of breathing* and *perception of breathing*. The *Dynamics* category refers to a subjective feeling of inner movement that is characterized by a certain *speed*, *direction* and *quality*. The experience of *Relaxation* is perceived as particularly deep and can be classified in the category *depth*.

The main category of FEELING can be broken down into three subcategories: *Intensity*, *Evaluation* and *Physical Localization* of the experience. The participants report emotions of varying *Intensity*, which are described as either *low* or *high*. The emotional experience is evaluated in the dimensions of *positivity*, *negativity* or *neutrality*. In addition, the emotional experience during the ASC can also be associated with certain areas of the body. This is reflected in the category of *Physical Localization*.

The main category of MENTAL ACTIVITY during an ASC encompasses the following subcategories: *Thoughts*, *Inner Visual Phenomena*, *Orientation*, *Attention*, *Ability to Act* and *Alertness*. They can be subdivided as follows: *Thoughts* change during ASC in terms of their thematic *content*, their *clarity* and their *mental dynamics*, which relate to the inner movement of thought. The participants perceive *luminosity*, *images* or *colors*, which represent different aspects of *Inner Visual Phenomena*. In connection with *Orientation*, the participants express an altered perception of *time* and *space* or being mentally present in the *here* and *now*. *Attention* changes in such a way that the *focus* shifts and there is a *loss of the ability to select*. This means that the ability to select internal and external stimuli in a targeted manner diminishes. One’s own *Ability to Act* is also perceived differently, both in terms of *passive* influence and *active* experience. The degree of *Alertness*, i.e. how awake or sleepy a person feels, is also recorded as a separate characteristic.

### Phase 3: ASC, body

Figure 5 shows the body-related dimensions of *Phase 3: ASC* under the overarching main category of BODY. This is divided into the four subcategories of *Bodily Sensations*, *Breathing*, *Dynamics* and *Relaxation*.

The following sub-sub-categories can be identified within the *Bodily Sensations* category: *Loss of bodily sensations*, *sensitivity*, *connection with surrounding environment*, *temperature* as well as *weight* and *decrease in body boundaries*. The subcategory *loss of bodily sensations* occurs nine times and has therefore been highlighted in color in Fig. 5 (marked in yellow). A reduced or completely eliminated body sensation is experienced as *pleasant* by the participants in their *affective perception* (2, line 451; 8, line 556). It is expressed, among other things, by the *absence of pain* (*painless*: line 535, 8; line 396, 12). In addition, the participants report that they are no longer consciously aware of their own body in these states. These experiences are recorded in the subcategory *decrease in body awareness* (line 470, 3; line 912, 4; line 563, 9; line 440, 10; line 490, 11; line 177, 13).

“*Um*,* (…) and what I found amazing was that I didn’t even notice the other (areas of my body)*,* well*,* I don’t know*,* the stomach or whatever. And that was somehow also very pleasant. So not feeling the whole thing anymore*,* so to speak*,* but that it melted away*.” (participant 2, line 451).

The ASC can lead to changes in somatosensory perception, with altered *sensitivity* being described in particular. This is perceived in *quality* as a *tingling* sensation (line 496, 2; line 287, 3; line 417, 13), which is *localized* in various regions of the body, including the *chest* (line 316, 3; line 417, 13), *legs* (line 322, 3) and *hands* (line 496, 2). With regard to the perceived boundaries of the body, a *connection with surrounding environment* is experienced, with most people reporting a connection with the *water* (line 475, 2; line 523, 3; line 456, 12). This experience is accompanied by a dissolution of body boundaries, so that the boundary between one’s own body and the water is perceived as permeable or blurred. In one individual case, a similar experience is also described in relation to the surrounding *air* (line 456, 12). The subjectively perceived *temperature* of the body is experienced as *warm* (line 404, 3; line 505, 4; line 543, 8; line 394, 12). In addition, a change in the perceived *weight* of the body can occur. This is perceived either as physical *heaviness* (line 483, 9; line 135, 12) or as *lightness* (line 494, 9). Furthermore, several test subjects express a *general decrease in body boundaries* (line 307, 2; line 398, 3; line 912, 4; line 214, 13).

In the course of the ASC, there are striking modifications to *Breathing*, particularly in relation to the *depth* and *perception of breathing*. The subcategory *depth of breathing* describes *deep* breathing (line 553, 6; line 512, 8; line 106, 13). In addition, there is an altered *perception of breathing*, which can consist of either an *increased* (line 479, 3; line 175, 6; line 432, 8) or *decreased perception*. The latter can go so far that a complete *loss of breath perception* is described (line 135, 7; line 526, 9).

The *Dynamics* category highlighted in yellow in Fig. 5 is differentiated into the dimensions of *speed*, *direction* and *quality* of the experienced movement. The perceived *speed* of movement varies between *slow* (line 702, 4; line 386, 12) and *fast* (line 393, 13). The participants report different *directions* of movement: A dynamic directed *downwards* (line 259, 1; line 665, 8; line 386, 12), *outwards* (line 167, 2; line 523, 3; line 50, 13) or *inwards* (line 523, 3; line 668, 4). The *quality* of dynamism encompasses various phenomena: states of *immobility* (line 476, 9; line 265, 12), a subjectively perceived *sinking* (line 222, 1; line 685, 4; line 99, 12; line 123, 13), the experience of an external *pull* or the feeling of *being pulled* (line 654, 10; line 406, 12; line 123, 13), *rotational movements* of the body (line 828, 4; line 582, 10) and an inner feeling of *expansion* (line 167, 2; line 523, 3; line 239, 13).

“*But not really as if I was falling downwards in space*,* but rather as if I was falling downwards inside myself. Before that*,* it was already very spatial. And then it felt like I was staying in the same place*,* but I was just falling into myself*.” (participant 4, line 668).

“*(…) In the meantime*,* I also had the feeling that I was going back and forth*,* more or less in the water. (4) Like a puddle in the water that spreads in and out and-*,* (…) again with the breath-*,* (6) yes*,* and then I simply enjoyed this feeling immensely*.” (participant 3, line 523).

The *Relaxation* category is an important characteristic of the ASC. The participants perceive the ASC state as *deep* relaxation (line 226, 6; line 158, 7; line 493, 8; line 483, 9; line 110, 11; line 99, 12; line 54, 13; line 268, 14). In addition, a muscular relaxation is described. This *releases* previously existing muscular *tension* (line 677, 1; line 146, 9; line 394, 12; line 472, 13).

“*Still through this heaviness*,* muscular too. Or muscular relaxation too. I didn’t have any pain or tension anywhere. Instead*,* my muscles were really soft. I felt really soft. And a feeling of warmth in my entire body. No tension rather something open. (…) Relaxed*,* physically too*,* yes*.” (participant 12, line 394).

### Phase 3: ASC, feeling

The emotional dimensions of *Phase 3: ASC* with the central category of FEELING are broken down into the subcategories of *Intensity*, *Evaluation* and *Physical Localization* (see Fig. 6).

A *low* emotional *Intensity* is associated with *not* experiencing anything *emotionally intense* (line 740, 1; line 765, 2; line 445, 11; line 440, 12), whereas *intense feelings* are reported when the intensity is *high* (line 416, 3; line 448, 6; line 485, 14). The subcategory *Evaluation* serves to classify the subjective emotional quality of the ASC experience by the participants. A distinction is made between perceived *positivity*, *negativity* and *neutrality*.

Positive evaluations are cited more than seven times and were therefore visually highlighted in yellow. The positive emotions include *joy* and *happiness* (line 436, 6; line 463, 13; line 434, 14), the experience of *freedom from fear* (line 403, 3; line 720, 4), a feeling of *safety* (line 565, 1; line 438, 3; line 303, 6; line 371, 12) and a frequently reported feeling of *security* (line 740, 1; line 765, 2; line 294, 3; line 720, 4; line 191, 6; line 599, 8; line 516, 9; line 371, 12). In addition, the *feeling* of *being enveloped* (line 721, 1; line 720, 4; line 599, 8), experiences of *connection* (line 475, 2; line 416, 3; line 191, 6) and a state of general *contentment* (line 740, 1; line 167, 6; line 183, 14) were also described. In the negativity category, two participants expressed a feeling of *slight anxiety* (line 377, 2; line 371, 12). In addition, the *neutrality* category provides information on a neutral emotional evaluation of the ASC experience, with the participants reporting *nothing emotionally intense* (line 740, 1; line 444, 12) or classifying the experience as *neither negative nor positive* (line 352, 9; line 316, 13). The subcategory *Physical Localization* includes information on the localization of the experienced emotions in the body. The sensations are often located in the *middle of the body* (line 424, 3), in the area of the *heart* (line 804, 4; line 360, 6; line 465, 13), in the *upper body* (line 804, 4), in the *abdomen* (line 226, 3; line 361, 6) and in the *head* (line 226, 3).

“*(5) I don’t know for how long*,* but for a while there was no fear (…)*,* such detachment*,* (…) freedom and connection at the same time. (…) That then (…)*,* yes*,* that also gave me strength somehow. (…) And now I still feel like*,* like recharged*.” (participant 3, line 416).

### Phase 3: ASC, mental activity

The various aspects of MENTAL ACTIVITY during *Phase 3: ASC* are shown in Fig. 7. The central subcategories include *Thoughts*, *Inner Visual Phenomena*, *Orientation*, *Attention*, *Ability to Act* and *Alertness*. The following section details the category of *Thoughts*, which can be broken down into the subcategories of *content*, *clarity* and *mental dynamics*.

The frequently described *content* (yellow marking of the category) of *Thoughts* comprises various mental phenomena, including autobiographical *memories* (line 295, 3; line 828, 4; line 261, 13), *images* (line 359, 3; line 249, 9; line 620, 10; line 348, 12; line 31, 13) and *meta-reflections* (line 589, 1; line 896, 8; line 383, 11). With regard to the *clarity* of thoughts, *concrete*, clearly identifiable content can be identified (line 425, 8), but also *abstract*, elusive thought processes that *cannot be narrated* by the participants (line 598, 7; line 425, 8; line 358, 11; line 57, 12).

In many instances, the *mental dynamics* during the ASC phase are characterized by reduced, i.e. *calm*, cognitive activity (yellow marking of the category). This can be seen, for example, in the *absence of mental content* (line 696, 1; line 928, 4; line 514, 7; line 559, 8) or a *decrease in thoughts* (line 600, 1; line 479, 3; line 315, 6; line 292, 7; line 361, 12; line 288, 13; line 528, 14). This experience is often accompanied by a subjective *calm* or *mentally relaxed* state (line 413, 3; line 440, 11; line 371, 12). In one individual case, however, a *lively mental dynamic* was also reported, which is characterized by *associative* cognitive content (line 425, 8).

During *Phase 3: ASC*, the participants report on *Inner Visual Phenomena*, which are divided into the subcategories of *luminosity*, *images* and *colors*. The term *luminosity* describes perceptions of radiant or luminous phenomena. The following *contents* can be experienced in this context: Perceptions such as a luminous *layer of energy around the body* (line 804, 2; line 599, 8), *radiant images* (line 392, 9), *points of light* (line 316, 3) or a *radiant body* (line 451, 1; line 420, 13; line 439, 14) are described. The subcategory *localization* indicates that these visual impressions are partly located *outside the body* (line 470, 3; line 804, 2; line 559, 8). These phenomena occur in a *bright* or *white color* (line 333, 3; line 370, 9; line 426, 13). In addition to *luminosity*, inner *images* frequently occur (line 359, 3; line 152, 6; line 141, 7; line 559, 8; line 51, 9; line 620, 10; line 348, 12; line 28, 13), which vary in terms of their *clarity* and *vividness*. The image perceptions are predominantly perceived as *unclear* and *diffuse* (line 466, 9; line 633, 10; line 480, 14), but in some cases as distinctly r*eal* and *clear* (line 138, 9; line 622, 10). The *vividness* of the inner images can be very pronounced, with *associative* and *intense* visual content being experienced in particular (line 559, 8; line 150, 9). In addition, visual impressions in the form of *colors* also occur (line 333, 3; line 1028, 4; line 873, 8; line 440, 9).

“*Yes*,* yes. And somehow*,* (…) something was also bright. So in*,* in this open forehead area somehow*,* it was also a bit*,* well*,* or if I put myself in there again now. So not just pulled up in the dark like that*,* but very*,* very much as if it were a ray of light or […]*.” (participant 13, line 420).

In the *Orientation* category, changes in the perception of *time* and *space* as well as the subjective experience of presence in the *here and now* are reported. Specific subcategories can be identified that reflect different aspects of temporal orientation: *Passage of time*, *loss of sense of time* and *duration of time*. With regard to the subjective *passage of time*, i.e. the perceived speed of the passage of time, participants often report a perceived *slowing down* of time (line 720, 4; line 307, 12). A particularly frequently described phenomenon is the *loss of the sense of time* (line 65, 3; line 139, 7; line 559, 8; line 319, 9; line 468, 10; line 265, 12), which in some cases comes to a complete *standstill of time* (line 964, 1; line 556, 8). In the subcategory *duration of time*, the tendency to *overestimate the time* that has actually elapsed is recorded (line 1073, 4; line 139, 7; line 250, 12), whereby the duration of the ASC experience is subjectively perceived as longer than it objectively is. The category *here and now* describes an intense experience of *mental presence* in the current moment and in relation to the surroundings (line 411, 1; line 448, 6; line 486, 10; line 300, 13; line 268, 14).

In addition to temporal changes, there are also disturbances in *spatial* orientation. In several cases, participants report a *loss of spatial orientation* (line 415, 1; line 167, 2; line 134, 7; line 546, 8; line 253, 12; line 637, 14).

“*Yes. No*,* it’s more of a very pleasant feeling*,* so it’s more (…)*,* so this realization*,* ah*,* I*,* I hardly feel my body*,* or I*,* um*,* it’s motionless*,* I don’t move. Nothing moves*,* even time doesn’t move.*” (participant 8, line 599).

The *Attention* category is divided into the subcategories of *focus* and *loss of the ability to select*. Several participants reported that the *focus* of attention during the ASC was *undirected*, i.e. without a conscious goal or intended focus. This form of directing attention is described as a *bottom-up* controlled process in which only that which spontaneously enters consciousness at the respective moment is perceived (line 451, 1; line 528, 2; line 380, 3; line 782, 4; line 627, 164, 6; line 518, 8; line 327, 12; line 334, 13). Another phenomenon is the *alternation* of attention *between body and mind* (line 481, 3; line 448, 6; line 697, 8; line 56, 9; line 530, 10). Attention oscillates between the perception of physical sensations and the focus on inner thoughts or mental content. In addition, there may be a *loss of the ability to select* (line 828, 4; line 615, 7; line 319, 12). This manifests itself in a limited ability to focus on or consciously select specific content.

“*Well*,* it was very diffusely distributed. Well*,* I can’t say that I directed my attention to anything in particular or that it was focused on anything in particular*.” (participant 4, line 782).

The majority of participants describe the category *Ability to Act* in this state as *passive* (line 754, 1; line 295, 4; line 648, 8; line 352, 9; line 340, 12), the absence of being able to act or react, experience happened. Only in individual cases, an *active* ability of movement is also perceived (line 677, 1; line 648, 8).

The final cognitive category, *Alertness*, refers to the subjective experience of *being fully awake* during the ASC (line 241, 6; line 596, 7; line 873, 8; line 217, 13; line 529, 14).

## Discussion

The present qualitative study provides, for the first time, a micro-phenomenological analysis of ASC in the context of Floatation-REST, offering a detailed understanding of the subjective experience during a float-induced ASC episode.

Our core finding is a four-phase of ASC with a clearly delineated macrostructure. However, the detailed analysis of *Phase 2: Transitional Phase* revealed a more complex and individualized transitions process. Phase 2 could be subdivided into three distinct but variably ordered subphases:

*2 A: Loss of Orientation*, characterized by disorientation along temporal and spatial dimensions, *2B: Altered Body Perception* characterized by a decrease in body awareness and a dissolution of bodily boundaries, accompanied by a positively connoted experience of this phenomenon.

*2 C: Letting Go*, characterized by mental release and deepening relaxation, (*see* Fig. 5). One participant (no. 6, see Fig. 3), moved directly from *Phase 1: Initial Phase* to the ASC without a clear transitional phase. This heterogeneous sequential structure may be partially explained by Poynter’s [49] time-memory model, which posits that retrospectively, less meaningful or routine experiences, such as transitional phases, are remembered with less clarity than intense or extraordinary events like the ASC itself. Alternatively, the differences may reflect individual attentional focus directed either toward orientation, altered bodily sensations, or the subjective experience of letting go.

Subphase *2 C: Letting Go* emerged as a central mechanism of transition. Participants described a process of mental release that coincided with increasing relaxation. This process appears to intensify the transition into ASC. It raises the question: Is the ability to “let go” a prerequisite for entering ASC—or merely one potential route among others? This question opens parallels with meditation research. In the field of meditation, mental letting go is also regarded as a significant process for attaining deeper ASC [15, 44]. In a micro-phenomenological study conducted by Petitmengin et al. [44] mental letting go emerged as a crucial component, wherein individuals first detach from their immediate situation, and their attention oscillates between emerging thoughts and a return to present-moment awareness. This process often coincides with a diminished sense of bodily self-awareness. This phenomenon is mirrored in Subphase *2B: Altered Body Perception*, in which participants report a dissolution of bodily boundaries. According to Linares Gutiérrez et al. [13], such boundary-dissolving experiences are also considered core features of meditative states, which have been corroborated by a further micro-phenomenological investigation by Nave et al. [15], which also identifies the act of letting go as a central catalyst for the dissolution of self-boundaries.

The synchronic analysis offers a nuanced phenomenological account of ASC induced by Floatation-REST. On the bodily level, key experiential features include a diminished perception of physical sensations, an internally perceived sense of dynamic movement, and a profound state of relaxation (see Fig. 5). Emotionally, the ASC is predominantly experienced as positive, with participants frequently reporting feelings of safety, security, and joy (see Fig. 6). The positive experience of the ASC could also be related to the ability to get involved in the experience or to let go, as is the case in the previous sub-phase *2 C: Letting go*. On the cognitive level, although thoughts are present during the ASC, their intensity diminishes over time, giving rise to a markedly quieter mental landscape that may evolve into a nearly content-free state of awareness (see Fig. 7). Such decrease of mind wandering is also reported in meditation proficient individuals while meditating [50, 51]. Additionally, participants describe visual phenomena, such as inner imagery and experiences of luminosity. Attention during ASC is largely undirected and primarily shaped by spontaneously emerging stimuli (i.e., bottom-up processes). Participants often report a perceptual fluctuation between bodily and mental sensations. Goal-directed thought recedes into the background, making way for open and receptive modes of awareness in which internal and external impressions are perceived in an unstructured and spontaneous manner. A further defining characteristic of the ASC experience is a pronounced loss of temporal perception, with participants frequently reporting a disrupted or absent sense of time (see Fig. 7). The fading of the sense of self and the sense of time are two core features of ASC [18, 19] reported by our participants, that may carry therapeutic relevance. People with depression, anxiety and other mental health issues frequently experience an intensified distressing awareness of both time and self [21]. Floatation-REST temporarily downregulates and even suspends these, which could be key to understanding its therapeutic potential and that of other interventions linked to ASC such as meditation and psychedelics.

Our findings show notable convergence with phenomenological accounts of deep meditative states as described by Metzinger [52]. In both contexts, participants reported the emergence of profound positive emotional states. Notably, the occurrence of luminous phenomena often regarded as characteristic of advanced meditative experiences was documented in Floatation-REST. While prior research by Kjellgren et al. [11] identified basic visual impressions such as fleeting sparks of light, our study revealed significantly more complex visual phenomena during Floatation-REST sessions. These included rare and vivid experiences such as the perception of a luminous envelope surrounding the body. Such elaborate perceptual events may be associated with the participants’ contemplative disposition: Four of the seven individuals who reported luminosity practiced meditation on a daily or weekly basis, while the remaining three engaged in such practices at least monthly. Further corroboration is provided by a qualitative study conducted by Costines et al. [53], which identified experiences of timelessness and disembodiment as core characteristics of meditative states in addition to the phenomenal quality of luminous clarity.

In comparison with the broader body of literature, Floatation-REST appears to reliably induce ASC that mirror the phenomenology of meditative states. However, there is a key methodological difference: Whereas studies on ASC during meditation predominantly involved highly experienced practitioners, the present Floatation-REST study did not require participants to have prior experience with floating or meditation. However, participants who generally express interest in a Floatation-REST study may exhibit an openness toward contemplative experiences. That is why we found so many interested participants with prior contemplative experience. Our sample is thus far from representative. That is, individual participants may have a greater awareness of their inner processes and potentially could report experiences in a more nuanced way. Our findings suggest that Floatation-REST provides an accessible way to enter deep ASC that usually require advanced meditative training.

Another difference between meditation and floatation-REST lies in the process of accessing ASC. While floatation-REST facilitates a physical state of deep relaxation (bottom-up) that then leads to ASC, ASC in meditation may be accessed through a more top-down route that then leads to physical relaxation.

The potential for clinical application of Floatation-REST may lie in exactly this feature: It offers access to deep states of relaxation in which thoughts and bodily sensations recede to people who do not have prior training or regular training in meditation. This may be particularly important for people with mental health conditions such as depression or people with chronic pain conditions. Daily meditation practice requires effort and discipline, which can be a threshold, especially for people with chronic conditions. Through its ability to quiet thought, soften bodily perception, and temporarily dissolve time structures Floatation-REST could offer a momentary relief from chronic suffering. These insights support future research into Floatation-REST as a therapeutic tool for mental health care, particularly for populations underserved by conventional approaches. Our sample included many individuals with contemplative experience (see Limitations), who may be more familiar with and comfortable engaging in inward-directed attention. Consequently, our findings on the induction of altered states of consciousness through flotation may not fully generalize to clinical populations. Under conditions of markedly reduced external stimulation, some patients may be confronted more directly with depressive rumination or other distressing inner experiences, which could in some cases heighten psychological vulnerability. This underscores the importance of implementing appropriate safeguards for vulnerable participants, such as allowing them to terminate the session or turn on the lights at any time. At the same time, existing empirical literature indicates that individuals with anxiety and depression can benefit substantially from floatation-based interventions [6].

### Limitations

The results should be interpreted with the understanding that the categorical distinction between phases and between physical, emotional, and mental aspects represents a methodological simplification. In actual experience, these dimensions cannot be strictly separated. Rather, they are closely interwoven and mutually influential processes that interact with the environment [54]. The example of Floatation-REST illustrates the extent to which cognitive processes are embedded in bodily-sensory perceptions, thereby highlighting the fundamental unity of body, mind, and environment in human experience [55]. The data analysis was conducted by the interviewer, which may be regarded as a methodological limitation. At the same time, the interviewer’s in-depth familiarity with the subject matter facilitated a nuanced interpretation of the data. Reliability of the analysis was ensured by reflecting each step of the analysis in the team.

Describing experiences of ASC presents a challenge and deviates considerably from everyday modes of communication [56]. The analysis revealed varying degrees of data saturation depending on the specific content of the ASC: mental activity was described in the greatest detail, whereas physical sensations and emotions were articulated less extensively. This discrepancy may be attributable to limitations in expressive capacity. Other micro-phenomenological studies (e.g. Schmidt et al. [57]) have therefore deliberately recruited verbally adept participants with meditation experience. In contrast, the present study intentionally included participants without prior experience, which may have resulted in less precise verbal accounts.

To ensure interpretative richness (or ‘thickness’), even infrequently mentioned categories were incorporated into the semantic networks. In micro-phenomenological analysis, even rarely occurring themes are considered relevant, as they can reveal subtle or unconscious content and contribute to the overall density and depth of the data [58].

A common critique of micro-phenomenological methods concerns the potential for cognitive distortion in retrospective reports [59]. In the present study, such memory distortion was minimized by conducting the interviews immediately following the Floatation-REST session. Particularly salient or unusual experiences, such as ASC, are known to be well retained in memory (Poynter, 1983). The “evocation” technique employed at the beginning of the interviews served to activate episodic memory through sensory re-experiencing processes, thereby supporting authentic and valid recall [59, 60]. Micro-phenomenological indicators of successful recall include among others slowed speech, pauses, and detailed descriptions accompanied by gestures [59], which were evaluated using a interview quality checklist prior to analysis.

A potential influence of prior experience and expectations on the experience of ASC warrants discussion. Whereas prior float experience ranged from one to five sessions, a substantial number of participants had extensive contemplative practice, which may limit the generalizability of the float-related findings to the broader population. We relied on a typical convenience sample and did not specifically recruit individuals with contemplative experience. In a university city—and particularly in Freiburg—many people practice meditation. Such individuals are also more likely to be interested in trying and participating in a flotation study. However, Norlander et al. [12] found no effect of contemplative background on ASC experiences, the present study similarly supports this finding: All participants, regardless of prior experience, reported ASC phenomena.

Participants’ expectations regarding the Floatation-REST session were not assessed in this study, which could be addressed in future research. A possible concern is that some experiences reported during flotation could be influenced by increased suggestibility or self-suggestibility brought on by the environment. This underscores a broader issue in altered states of consciousness research: separating true effects of an intervention from those shaped by expectations or contextual factors.

## Conclusion

The phase model developed as part of the present study provides a differentiated approach to the inner experience during ASC and could serve as a foundation for individually tailored therapeutic approaches in the future. Our micro-phenomenological findings regarding ASC hold significant relevance for the development of future clinical applications of Floatation-REST, particularly in the treatment of mental health conditions such as anxiety and depression [6, 61] as well as concerning the induction and assessment of non-ordinary experiences [62]. Future research could systematically examine how silence, darkness, and reduced gravity affect the floatation experience, particularly in relation to ASC and therapeutic outcomes [63].

## Data Availability

The quotations (participant, line number) from each semantic network of the ASC (Figs. 5, 6 and 7) and the micro-phenomenological interview quality checklist are available in the OSF repository: https://osf.io/3v9zc/. Further data are available from the corresponding author on reasonable request.

## References

[1] Lashgari E, Chen E, Gregory J, Maoz U. A systematic review of flotation-restricted environmental stimulation therapy (REST). BMC Complement Med Ther. 2025;25:230. 40611079 10.1186/s12906-025-04973-0 PMC12224670

[2] Suedfeld P, Borrie RA. Health and therapeutic applications of chamber and flotation restricted environmental stimulation therapy (REST). Psychol Health. 1999;14(3):545–66.

[3] Van Dierendonck D, Te Nijenhuis J. Flotation restricted environmental stimulation therapy (REST) as a stress-management tool: A meta-analysis. Psychol Health. 2005;20(3):405–12.

[4] Bood SA, et al. Eliciting the relaxation response with the help of flotation-REST (restricted environmental stimulation technique) in patients with stress-related ailments. Int J Stress Manag. 2006;13(2):154.

[5] Kjellgren A, Sundequist U, Norlander T, Archer T. Effects of flotation-REST on muscle tension pain. Pain Res Manag. 2001;6(4):181–9. 11854763 10.1155/2001/768501

[6] Feinstein JS, Khalsa SS, Yeh HW, Wohlrab C, Simmons WK, Stein MB, et al. Examining the short-term anxiolytic and antidepressant effect of Floatation-REST. PLoS ONE. 2018;13(2):e0190292. 10.1371/journal.pone.0190292 PMC5796691 29394251

[7] Jonsson K, Kjellgren A. Promising effects of treatment with flotation-REST (restricted environmental stimulation technique) as an intervention for generalized anxiety disorder (GAD): a randomized controlled pilot trial. BMC Complement Altern Med. 2016;16:1–12. 27016217 10.1186/s12906-016-1089-x PMC4807536

[8] Khalsa SS, Moseman SE, Yeh HW, Upshaw V, Persac B, Breese E, et al. Reduced environmental stimulation in anorexia nervosa: An early-phase clinical trial. Front Psychol. 2020;11:567499. 33123048 10.3389/fpsyg.2020.567499 PMC7573249

[9] Hruby H, Schmidt S, Feinstein JS, Wittmann M. Induction of altered states of consciousness during Floatation-REST is associated with the dissolution of body boundaries and the distortion of subjective time. Sci Rep. 2024;14(1):9316. 38654027 10.1038/s41598-024-59642-y PMC11039655

[10] Kjellgren A, Sundequist U, Sundholm U, Norlander T, Archer T. Altered consciousness in flotation-REST and chamber REST: Experience of experimental pain and subjective stress. Soc Behav Pers Int J. 2004;32(2):103–15.

[11] Kjellgren A, Lyden F, Norlander T. Sensory isolation in flotation tanks: Altered states of consciousness and effects on wellbeing. Qual Rep. 2008;13:636–56.

[12] Norlander T, Kjellgren A, Archer T. The experience of flotation-REST as a function of setting and previous experience of altered state of consciousness. Imagination Cogn Pers. 2000;20:161–78.

[13] Linares Gutiérrez D, Schmidt S, Meissner K, Wittmann M. Changes in subjective time and self during meditation. Biology. 2022;11(8):1116. 35892973 10.3390/biology11081116 PMC9330740

[14] Millière R. Looking for the self: phenomenology, neurophysiology and philosophical significance of drug-induced ego dissolution. Front Hum Neurosci. 2017;11:191130. 10.3389/fnhum.2017.00245 PMC5441112 28588463

[15] Nave O, Trautwein FM, Ataria Y, Dor-Ziderman Y, Schweitzer Y, Fulder S, et al. Self-boundary dissolution in meditation: A phenomenological investigation. Brain Sci. 2021;11(6):819. 10.3390/brainsci11060819 PMC8235013 34205621

[16] Preller KH, Vollenweider FX. Phenomenology, structure, and dynamic of psychedelic states. Behav Neurobiol psychedelic drugs. 2018;36:221–56. 10.1007/7854_2016_459 28025814

[17] Timmermann C, Bauer PR, Gosseries O, Vanhaudenhuyse A, Vollenweider F, Laureys S, et al. A neurophenomenological approach to non-ordinary states of consciousness: hypnosis, meditation, and psychedelics. Trends Cogn Sci. 2023;27(2):139–59. 36566091 10.1016/j.tics.2022.11.006

[18] Wittmann M. Modulations of the experience of self and time. Conscious Cogn. 2015;38:172–81. 26121958 10.1016/j.concog.2015.06.008

[19] Wittmann M. Altered states of consciousness: Experiences out of time and self. Cambridge, MA: MIT Press; 2018.

[20] Wittmann M, et al. Effects of psilocybin on time perception and temporal control of behaviour in humans. J Psychopharmacol. 2007;21(1):50–64. 16714323 10.1177/0269881106065859

[21] Vogel DH, Krämer K, Schoofs T, Kupke C, Vogeley K. Disturbed experience of time in depression—evidence from content analysis. Front Hum Neurosci. 2018;12:66. 29515385 10.3389/fnhum.2018.00066 PMC5826190

[22] Giommi F, Bauer PR, Berkovich-Ohana A, Barendregt H, Brown KW, Gallagher S, et al. The (In) flexible self: Psychopathology, mindfulness, and neuroscience. Int J Clin health psychology: IJCHP. 2023;23(4):100381. 10.1016/j.ijchp.2023.100381 PMC10033904 36969914

[23] Fuchs T. Depression, intercorporeality, and interaffectivity. J Conscious Stud. 2013;20(7–8):219–38.

[24] Elkjær E, Mikkelsen MB, Michalak J, Mennin DS, O’Toole MS. Motor alterations in depression and anxiety disorders: A systematic review and meta-analysis. J Affect Disord. 2022;317:373–87. 36037990 10.1016/j.jad.2022.08.060

[25] Carhart-Harris RL, Goodwin GM. The therapeutic potential of psychedelic drugs: past, present, and future. Neuropsychopharmacology. 2017;42:2105–13. 28443617 10.1038/npp.2017.84 PMC5603818

[26] Balzani C, Naudin J, Vion-Dury J. Phénoménologie expérientielle de l’écoute musicale en psychiatrie [Experiential phenomenology of musical listening in psychiatry]. Annales Médico-psychologiques, revue psychiatrique. Elsevier; 2014. pp. 524–9.

[27] Bourvis N, Vion-Dury J. Phénoménologie expérientielle de l’algie vasculaire de la face (AVF) [Experiential phenomenology of vascular facial fatigue (AVF)]. Annales Médico-psychologiques, revue psychiatrique. Elsevier; 2014. pp. 247–52.

[28] Cavaletti F, Heimann K. Longing for tomorrow: phenomenology, cognitive psychology, and the methodological bases of exploring time experience in depression. Phenomenology Cogn Sci. 2020;19(2):271–89.

[29] Depraz N, Gyemant M, Desmidt T. A first-person analysis using third-person data as a generative method. Construc Found. 2017;12:190–203.

[30] Petitmengin C, Navarro V, van Quyen M. Anticipating seizure: pre-reflective experience at the center of neuro-phenomenology. Conscious Cogn. 2007;16(3):746–64. 17590351 10.1016/j.concog.2007.05.006

[31] Petitmengin C. A neurophenomenological study of epileptic seizure anticipation. Handb phenomenology Cogn Sci. 2010;471–99.

[32] Valenzuela Moguillansky C, O’Regan JK, Petitmengin C. Exploring the subjective experience of the rubber hand illusion. Front Hum Neurosci. 2013;7:659. 24167480 10.3389/fnhum.2013.00659 PMC3805941

[33] Balas-Chanel A. La pratique réflexive. Un outil de développement des compétences infirmières [Reflective practice: a tool for developing nursing skills]. Issy-les-Moulineaux: Elsevier Masson; 2013.

[34] Gould C, Froese T, Barrett AB, Ward J, Seth AK. An extended case study on the phenomenology of sequence-space synesthesia. Front Hum Neurosci. 2014;8:433. 25071498 10.3389/fnhum.2014.00433 PMC4080762

[35] Horwitz EB, Stenfors C, Osika W. Writer’s Block Revisited: A Micro-Phenomenological Case Study on the Blocking Influence of an Internalized Voice. J Conscious Stud. 2018;25(3–4):9–28.

[36] Ollagnier-Beldame M, Coupé CDM. Meeting you for the first time: Descriptive categories of an intersubjective experience. Constructivist Found. 2019;14(2):167–80.

[37] Petitmengin C, Bitbol M. Listening from within. J Conscious Stud. 2009;16(10–11):363–404.

[38] Quidu M, Favier-Ambrosini B. L’articulation des données en première et troisième personnes. De la genèse d’une méthodologie originale en Science du sport [The articulation of data in the first and third person. On the emergence of an original methodology in sports science]. Intellectica-La revue de l’Association pour la Recherche sur les sciences de la Cognition (ARCo); 2014.

[39] Remillieux A, Petitmengin C, Ermine JL, Blatter C. Knowledge sharing in change management: A case study in the French railways company. J Knowl Manage Pract. 2010;11(2):hal–00524414.

[40] Van-Quynh A. Intuition in mathematics: a perceptive experience. J Phenomenological Psychol. 2017;48(1):1–38.

[41] Petreca B, Baurley S, Bianchi-Berthouze N. How do designers feel textiles? In: International Conference on Affective Computing and Intelligent Interaction (ACII). IEEE; 2015. pp. 982–987.

[42] Petreca BB. An understanding of embodied textile selection processes & a toolkit to support them. Royal College of Art (United Kingdom); 2016.

[43] Vásquez-Rosati A. Body Awareness to Recognize Feelings. Constructivist Found. 2017;12(2):219–26.

[44] Petitmengin C, van Beek M, Bitbol M, Nissou JM. What is it like to meditate? Methods and issues for a micro-phenomenological description of meditative experience. J Conscious Stud. 2017;24(5–6):170–98.

[45] Sanders JW, Millière R, Daily ZG, Carhart-Harris R, Timmermann C. DMT micro-phenomenology. 2024. 10.1093/nc/niag015 PMC13366535 42454183

[46] Demšar E, Windt J. Studying dream experience through dream reports: Points of contact between dream research and first-person methods in consciousness science. Dreaming and memory: Philosophical issues. Springer; 2024. pp. 85–117.

[47] Petitmengin C. Describing one’s subjective experience in the second person: An interview method for the science of consciousness. Phenomenology Cogn Sci. 2006;5(3–4):229–69.

[48] Valenzuela-Moguillansky C, Vásquez-Rosati A. An analysis procedure for the micro-phenomenological interview. Constructivist Found. 2019;14(2):123–45.

[49] Poynter WD. Duration judgment and the segmentation of experience. Mem Cognit. 1983;11:77–82. 6855562 10.3758/bf03197664

[50] Feruglio S, Matiz A, Pagnoni G, Fabbro F, Crescentini C. The impact of mindfulness meditation on the wandering mind: a systematic review. Neurosci Biobehavioral Reviews. 2001;131:313–30. 10.1016/j.neubiorev.2021.09.032 34560133

[51] Winter U, LeVan P, Borghardt TL, Akin B, Wittmann M, Leyens Y, et al. Content-free awareness: EEG-fcMRI correlates of consciousness as such in an expert meditator. Front Psychol. 2020;10:3064. 32132942 10.3389/fpsyg.2019.03064 PMC7040185

[52] Metzinger T. Der Elefant und die Blinden: auf dem Weg zu einer Kultur der Bewusstheit: mit mehr als 500 Erfahrungsberichten über das reine Bewusstsein [The elephant and the blind: on the way to a culture of consciousness: with more than 500 testimonials on pure consciousness]. Berlin; 2023.

[53] Costines C, Borghardt TL, Wittmann M. The phenomenology of pure consciousness as reported by an experienced meditator of the Tibetan Buddhist Karma Kagyu tradition. Philosophies. 2021;6(2):50.

[54] Shapiro L. Embodied cognition. 2nd ed. Routledge; 2019.

[55] Newen A, De Bruin L, Gallagher S, editors. The Oxford handbook of 4E cognition. Oxford University Press; 2018.

[56] Costines C, Schmidt TT. Phenomenology of psychedelic experiences and psychedelic-associated distressing effects: quantifying subjective experiences. 2024. 10.1007/7854_2024_562 39739177

[57] Schmidt S, Bauer PR, Trautwein FM. Neurophenomenology in action: Integrating the first-person perspective into the Libet experiment. Mindfulness. 2024; 1–14.

[58] Sergi V, Hallin A. Thick performances, not just thick descriptions: The processual nature of doing qualitative research. Qualitative Res Organ Management: Int J. 2011;6(2):191–208.

[59] Petitmengin C. The validity of first-person descriptions as authenticity and coherence. J Conscious Stud. 2009;16(10–11):252–84.

[60] Cohen G, Conway MA. Memory in the real world. Psychology; 2007.

[61] Kjellgren A, Buhrkall H, Norlander T. Psychotherapeutic treatment in combination with relaxation in a flotation tank: Effects on burn-out syndrome. Qualitative Rep. 2010;15(5):1243–69.

[62] Huber A, Kjellgren A, Passie T. Hypnagogia, psychedelics, and sensory deprivation: the mythic structure of dream-like experiences. Front Psychol. 2025;16:1498677. 40417014 10.3389/fpsyg.2025.1498677 PMC12098477

[63] Schiffer K, Pfeifer E, Stolterfoth C, Wittmann M. Silence, darkness, and gravity: A qualitative analysis of individual experiences during Floatation-REST. Spiritual Care. 2025;14(3):263–77.

